# Rapid and Sensitive Detection of *Didymella bryoniae* by Visual Loop-Mediated Isothermal Amplification Assay

**DOI:** 10.3389/fmicb.2016.01372

**Published:** 2016-08-30

**Authors:** Xiefeng Yao, Pingfang Li, Jinghua Xu, Man Zhang, Runsheng Ren, Guang Liu, Xingping Yang

**Affiliations:** Institute of Vegetable Crops, Jiangsu Academy of Agricultural Sciences/Jiangsu Key Laboratory for Horticultural Crop Genetic ImprovementNanjing, China

**Keywords:** gummy stem blight, *Didymella bryoniae*, muskmelon, loop-mediated isothermal amplification, primer design

## Abstract

*Didymella bryoniae* is a pathogenic fungus that causes gummy stem blight (GSB) in Cucurbitaceae crops (e.g., cantaloupe, muskmelon, cucumber, and watermelon). GSB produces lesions on the stems and leaves, and can also be spread by seeds. Here, we developed a rapid, visual, and sensitive loop-mediated amplification (LAMP) assay for *D. bryoniae* detection based on sequence-characterized amplified regions (GenBank accession nos GQ872461 and GQ872462) common to the two random amplification of polymorphic DNA group genotypes (RGI and RGII) of *D. bryoniae*; ideal conditions for detection were optimized for completion in 45 min at 63°C. The sensitivity and specificity of the LAMP assay were further analyzed in comparison with those of a conventional polymerase chain reaction (PCR). The sensitivity of the LAMP assay was 1000-fold higher than that of conventional PCR with a detection limit of 0.1 fg μL^-1^ of targeted DNA. The LAMP assay could be accomplished in about 45 min, with the results visible to the naked eye. The assay showed high specificity in discriminating all *D. bryoniae* isolates from seven other fungal pathogens that occur in Cucurbitaceae crops. The LAMP assay also detected *D. bryoniae* infection in young muskmelon leaves with suspected early symptoms of GSB disease. Hence, the technique has great potential for developing rapid and sensitive visual detection methods for the *D. bryoniae* pathogen in crops and seeds. This method has potential application in early prediction of disease and reducing the risk of epidemics.

## Introduction

Gummy stem blight (GSB) is a highly prevalent disease on cucurbit crops (e.g., cantaloupe, muskmelon, watermelon, and cucumber) worldwide (Keinath, 2011; Li et al., 2015). The disease is caused by the ascomycete *Didymella bryoniae* (Fuckel) Rehm [anamorph *Phoma cucurbitacearum* (Fr.) Sacc.], synonym *Stagonosporopsis cucurbitacearum* (Fr.) Aveskamp et al. (2010), which produces necrotic spots on leaves and lesions on the vines and stems (Keinath et al., 1995; Somai et al., 2002; Keinath, 2014). *D. bryoniae* can also be spread by a low level of contaminated seeds (Somai et al., 2002; Sudisha et al., 2006; Keinath, 2011). GSB is one of the most important biotic constraints for muskmelon and watermelon production in moist environments such as plastic tunnels and greenhouses, leading to significant yield losses and a reduction in fruit quality in cucurbit crops in China (Wolukau et al., 2007; Li et al., 2015). *D. bryoniae* is well adapted to infect cucurbits, and can rapidly colonize the host tissue and reproduce abundantly in humid greenhouses. As reported previously, the pathogen has been found to move from infected source seedlings to adjacent seedlings in transplant greenhouses (Keinath, 1996, 2011). Thus, a low level of latently infected seedlings could potentially result in a major disease epidemic under favorable environmental conditions (Ling et al., 2010). Although cultural practices and fungicides play an important role in GSB management (Finger et al., 2014), they require diagnosis of the pathogen during early stages of disease development in cucurbit crop production. Thus, early diagnosis, sensitive and rapid detection of *D. bryoniae* is very important to limit the spread of GSB in plants being moved in greenhouses or in the field.

The most common methods currently used for the rapid detection of *D. bryoniae* are based on several molecular detection tools. These include conventional polymerase chain reaction (PCR), PCR-enzyme-linked immunosorbent assay (PCR-ELISA; Keinath et al., 2001; Somai et al., 2002), and magnetic-capture hybridization multiplex real-time PCR, but these are only specific for detection of isolates of *D. bryoniae* in the random amplified polymorphic DNA (RAPD) group (RG) I genotype (Somai et al., 2002; Ha et al., 2009). Recently, to obtain a reliable, sensitive, and broad-spectrum diagnostic method for *D. bryoniae* isolates of both genotypes (RGI and RGII), an improved real-time PCR assay that is capable of detecting *D. bryoniae* isolates regardless of their genotype was developed (Ling et al., 2010), but the primer set has not been used in a direct seedling healthy assay. Among the currently used PCR assays, a high background in PCR-ELISA limits its adaptability and usefulness in general disease diagnosis (Ling et al., 2010), and PCR-based diagnostic procedures still have several intrinsic disadvantages, including the requirements for expensive laboratory instrumentation and reagents, and for appropriate training and technical expertise, which are often not available in poorly resourced laboratories and in rural areas of developing countries (Francois et al., 2011; Duan et al., 2014a; Moradi et al., 2014; Shen et al., 2016). Thus, there is a need to develop a straightforward, sensitive, rapid, and cost-effective method for the early diagnosis and *in situ* testing of *D. bryoniae.* Notomi et al. (2000) developed a novel DNA amplification technique named loop-mediated isothermal amplification (LAMP) that can be an effective method to address deficiencies of PCR-based methods, overcoming common limitations of current diagnostic methods (Notomi et al., 2000; Mori et al., 2013; Niessen, 2015). LAMP can rapidly amplify nucleic acids under isothermal conditions with a set of four to six specially designed primers and the large fragment of *Bst* DNA polymerase, which undergoes strand displacement activity to amplify target DNA in less than 60 min (Notomi et al., 2000; Nagamine et al., 2002b). The entire procedure is not difficult to perform and requires only an isothermal instrument, such as a water bath or heating block (Nagamine et al., 2002b). Additionally, the LAMP reaction is thought to have a higher tolerance to inhibitory substances than many PCR-based assays (Francois et al., 2011). LAMP products can easily be visualized by gel electrophoresis or by measuring turbidity caused by a white precipitate of magnesium pyrophosphate (Notomi et al., 2000; Nagamine et al., 2002b). Moreover, it can be monitored with the naked eye by adding colorimetric indicators, such as calcein, which produces a green fluorescent signal if the LAMP reaction is positive (Zhang et al., 2014). With these advantages, the LAMP method has been widely used for detection of plant pathogens, such as viruses (Hadersdorfer et al., 2011; Peng et al., 2012), bacteria (Hodgetts et al., 2015), nematodes (Kang et al., 2015), oomycetes (Hansen et al., 2016), and fungi (Shen et al., 2016). In recent years, the LAMP-based assay has grown in popularity for the detection of many plant-pathogenic fungi (Niessen and Vogel, 2010; Denschlag et al., 2012; Niessen, 2015). Taking these advantages into account, in this study the LAMP method was adapted to investigate latent infection and the early stages of disease in field samples infected with *D. bryoniae*.

Although a very recent report has described a LAMP assay targeting the conserved RNA polymerase II RPB140 (*RPB2*) gene to detect *D. bryoniae* in cucurbit seeds (Tian et al., 2016), the detection sensitivity and efficiency were lower. In addition, the study focused only on detection of *D. bryoniae* infection in seed samples and not on infected samples in the field. The present study was undertaken to develop a LAMP assay for the detection of *D. bryoniae* based on targeting a sequence-characterized amplified region (SCAR; GenBank accession nos GQ872461 and GQ872462) of genomic DNA from the two genotypes (RGI and RGII) of *D. bryoniae*. The method was applied to detection of *D. bryoniae* from young muskmelon leaves with suspected early symptoms of GSB disease. The results demonstrated that this method is specific and efficient. This new LAMP assay will provide important reference data for monitoring and controlling GSB caused by *D. bryoniae*. Early and accurate detection of the causal agent (*D. bryoniae*) of GSB in cucurbit crops also could lead to reduced use of fungicides, thus benefiting the environment, and may reduce the risk of disease epidemics.

## Materials and Methods

### Ethics Statement

Our study does not involve human specimens or tissue samples, or vertebrate animals, embryos or tissues. In our study, *D. bryoniae* isolates were collected from its host muskmelon or watermelon. The field is not protected in any way. The field study did not involve endangered or protected species.

### Fungal and Culture Conditions

Five *D. bryoniae* isolates were obtained from muskmelon and watermelon plants that we collected from Jiangsu, Anhui, and Zhejiang provinces in east China. Seven other fungal pathogens of Cucurbitaceae crops were collected from muskmelon, watermelon, and gourd from Jiangsu province, and an *Ascochyta pinodes* isolate was obtained from a pea plant at Zhejiang province. The plant-pathogenic fungi used in this study, as well as their host, geographical origin, and RAPD group, are listed in **Table 1**. All isolates of fungal species were maintained in the collection of Jiangsu Academy of Agricultural Sciences. Fungal isolates were stored as monoconidial cultures grown on potato dextrose agar (PDA) plates (200 g potato, 20 g glucose, 15 g agar in 1 L water) at 25°C and were stored on PDA at 4°C. The sporulation and inoculum density of spores were prepared based on our previous study (Li et al., 2015).

**Table 1:** Fungi isolates used in the conventional PCR and LAMP assay.

Species^a^	RAPD group^b^	Origin		PCR product^c^	
		Host	Geographical	Conventional PCR	LAMP
*Didymella bryoniae* (DBJSJY2)	RGI	Muskmelon	Jiangsu	**+**	**+**
*Didymella bryoniae* (DBAHHF2)	RGI	Muskmelon	Anhui	**+**	**+**
*Didymella bryoniae* (DBZJNB5)	RGI	Muskmelon	Zhejiang	**+**	**+**
*Didymella bryoniae* (DBJSNJ60)	RGI	Watermelon	Jiangsu	**+**	**+**
*Didymella bryoniae* (DBZJNB7)	RGII	Watermelon	Jiangsu	**+**	**+**
*Ascochyta pinodes* ZJ-1	–	Pea	Zhejiang	-	-
*Colletotrichum orbiculare* NJ-1	–	Watermelon	Jiangsu	-	-
*Pythium paroecandrum Drechsler*	–	Gourd	Jiangsu	-	-
*Alternaria alternata* LH1401	–	Muskmelon	Jiangsu	-	-
*Fusarium verticillioide*	–	Gourd	Jiangsu	-	-
*Fusarium oxysporum f.sp. niveum* Race 0	–	Watermelon	Jiangsu	-	-
*Fusarium oxysporum f.sp. niveum* Race 1	–	Watermelon	Jiangsu	-	-
*Fusarium oxysporum f.sp. niveum* Race 2	–	Watermelon	Jiangsu	-	-

*^a^All isolates of fungi species were maintained in the collection of Jiangsu Academy of Agricultural Sciences.*

*^b^Based on the presence of a PCR product of the expected size for RAPD group assignments (RGI and RGII) using RG-specific SCAR primers (**Supplementary Figure S1**; Keinath et al., 2001; Babu et al., 2015).*

*^c^Note that presence (+) or absence (-) are based on the presence of a PCR or LAMP product of the expected size.*

### Reagents

*Bst* DNA polymerase was purchased from New England Biolabs (Beijing) Ltd (Beijing, China). Calcein was bought from Sigma-Aldrich Co. LLC (Sigma, USA). *Taq* DNA polymerase, MnCl_2,_ and dNTPs were purchased from TaKaRa Biotechnology (Dalian) Co., Ltd (Dalian, China). All other reagents were analytical grade and were purchased from Sinopharm Chemical Reagent Co., Ltd (Suzhou, China).

### Plant Materials

Muskmelon (*Cucumis melo* L. var. ‘Japanese Sweet Babe’) plants containing three true leaves were inoculated by spraying with the spore suspension until the leaves were completely wet. The plants were then incubated at 25°C in a misted plastic tunnel at 90–100% relative humidity, as described previously (Li et al., 2015).

### DNA Extraction

For DNA extraction, isolates were grown on dried filter paper disks on PDA plates for 7 days. Mycelia were then harvested as described previously (Ling et al., 2010). Muskmelon genomic DNA was extracted from the leaves. All genomic DNA was extracted using a DNeasy Plant Mini Kit (Qiagen, Valencia, CA, USA) according to the manufacturer’s instructions. The DNA was quantified using 1% (w/v) agarose gel electrophoresis and stored at -20°C for further use.

### LAMP Primer Design

**FIGURE 1:**
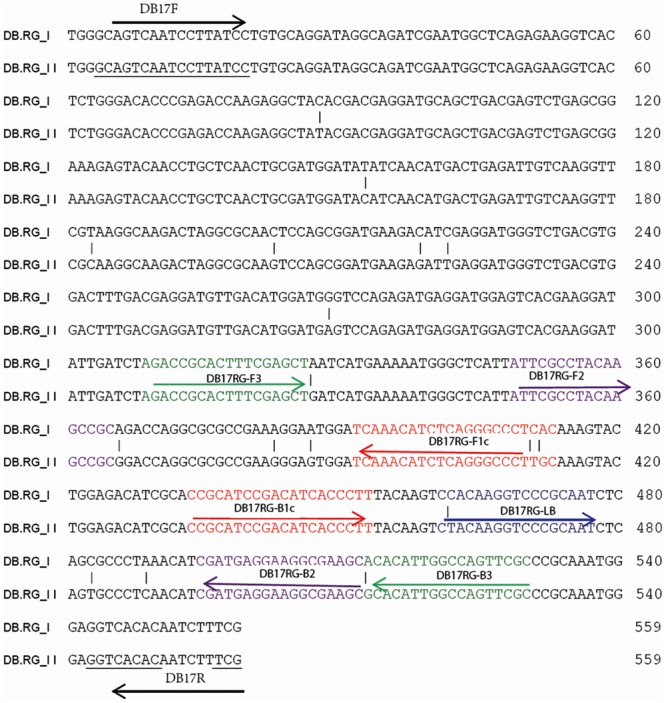
**Design of LAMP primers for detection of *D. bryoniae* (DB).** Schematic diagram of LAMP and conventional PCR primer binding sites within the alignment *D. bryoniae* RAPD marker sequence from RGI and RGII (GenBank accession nos GQ872461 and GQ872462; Ling et al., 2010) were used for this study. The sequences used for LAMP primers are indicated by different colors and arrows. FIP and BIP primers contain two distinct sequences: F1c plus F2 and B1c plus B2, respectively.

In a previous study, based on random amplification of polymorphic DNA (RAPD) markers, Ling et al. (2010) developed sequence characterized amplified regions (SCAR) primers (DB17 primer set) with broad-spectrum specificity that amplified a conserved sequence region common to both genotypes (RGI and RGII) of *D. bryoniae*. This SCAR, common to both genotypes of *D. bryoniae*, was identified in PCR products generated using the DB17 primer set (**Table 2**) (Ling et al., 2010). According to this conserved SCAR (GenBank accession nos GQ872461 and GQ872462), a set of LAMP primers, comprising two outer primers (DB17RG-F3 and DB17RG-B3), two inner primers (DB17RG-FIP and DB17RG-BIP), and one loop-backward primer (LDB17RG-LB) was designed using the LAMP primer software PrimerExplorer v.4^1^ (Eiken Chemical Co., Japan) (**Figure 1**). All of the oligomers were synthesized and purified by Beijing Genomics Institute (Shanghai, China). The designed primer sequences for *D. bryoniae* are shown in **Table 2**.

**Table 2:** Details of conventional PCR and LAMP primers used in this study.

Primer	Sequences (5′–3′)	Reference
Conventional PCR primers		
RG I-F	TGTCGTTGACATCATTCCAGC	Keinath et al., 2001; Somai et al., 2002
RG I-R	ACCACTCTGCTTAGTATCTGC	
RG II-F	GCTAAGCCTTAATCTAGCTGC	Keinath et al., 2001; Somai et al., 2002
RG II-R	GAGAGTAAGCTAACCTAAAGG	
DB17F	GCAGTCAATCCTTATCC	Ling et al., 2010
DB17R	CGAAAGATTGTGTGACC	
		
LAMP primers		This study
DB17RG-F3	AGACCGCACTTTCGAGCT	
DB17RG-B3	GCGAACTGGCCAATGTGT	
DB17RG-LB	TCCACAAGGTCCCGCAAT	
DB17RG-FIP	GTGAGGGCCCTGAGATGTTTGA	
(F1c plus F2)	ATTATTCGCCTACAAGCCGC	
DB17RG-BIP	CCGCATCCGACATCACCCTT	
(B1c plus B2)	GCTTCGCCTTCCTCATCG	

### Optimization of LAMP Reaction Conditions

The LAMP reaction was performed in a total volume of 25 μL. A visual fluorescent metal indicator (calcein) was added to the reaction mixture before amplification. For optimization of reagents, a range of concentrations of *Bst* DNA polymerase large fragment (2–8 U), dNTPs (2–10 mM), Mg^2+^ (2–8 mM), primers (2–4 μM), MnCl_2_ (0.1–1 mM), and calcein (2–8 μM) were evaluated. The LAMP reaction was performed in 0.2 mL microcentrifuge tubes in a water bath. Genomic DNA of *D. bryoniae* (strain DBJSJY2) as a template and ddH_2_O as a negative control were included in each assay. To identify the optimal reaction temperature and time for the visual detection of the LAMP reaction, the LAMP mixtures were incubated for 45 min at 61, 62, 63, 64, 65, 66, or 68°C in a water bath for 15, 30, 45, or 60 min. The reactions were terminated by heat inactivation at 80°C for 10 min. The reaction mixtures in the microcentrifuge tubes were visually inspected by the naked eye to determine the color change. Each product was confirmed by 2.0% agarose gel electrophoresis following staining with ethidium bromide (0.5 μg mL^-1^), and photographed under a UV transilluminator. There were three replications for each treatment, and the experiment was repeated twice.

### PCR Reaction

Assignment to the RGI and RGII genotypes of *D. bryoniae* strains in this study was performed by an RG-specific PCR according to previously published protocols (Keinath et al., 2001; Somai et al., 2002; Babu et al., 2015). In brief, PCRs were performed in a 25 μL reaction mixture containing 40 ng DNA, 2.0 μL 10x reaction buffer [50 mM Tris/HCl (pH 8.8), 1.5 mM MgCl_2_, 15 mM (NH_4_)_2_SO_4_ and 0.1% Triton X-100], 200 μM dNTPs, 2.5 U *Taq* DNA polymerase (5 U μL^-1^), and 0.25 μM each primer. The thermal cycling program was: 94°C for 2 min, and 35 cycles of 94°C for 1 min, 62°C for 1 min, and 72°C for 2 min (Babu et al., 2015). The DB17 primer set was used to amplify the conserved SCAR common to both genotypes of *D. bryoniae* (**Table 2**) (Ling et al., 2010). The DB17 primer set-based PCR was carried out with the following cycling conditions: 95°C for 2 min, and 35 cycles of 95°C for 1 min, 50°C for 45 s, and 72°C for 1 min (Ling et al., 2010). PCR amplifications were performed in a 25 μL reaction volume using Platinum^®^ *Pfx* DNA polymerase (Thermo Fisher Scientific Inc., Waltham, MA, USA). All of the primers were synthesized and purified by Beijing Genomics Institute (Shanghai, China). PCR products were detected by electrophoresis on a 2.0% (w/v) agarose gel containing ethidium bromide (0.5 μg mL^-1^) in 1x TAE buffer (pH 8.0) at 90 V for 1 h and were visualized under UV light.

### Specificity of the LAMP Assay

The specificity of the LAMP assay was verified with genomic DNA of both genotypes (RGI and RGII) of the *D. bryoniae* strains, seven other fungal pathogens found in Cucurbitaceae crops, and one fungal pea pathogen (*A. pinodes* anamorph: *D. pinodes*; **Table 1**), which is in the same genus as *D. bryoniae*. Optimal LAMP reaction components and conditions were used as described earlier. The LAMP assay was performed and assessed twice.

### Sensitivity of Detection between LAMP and Conventional PCR Assays

Template DNA from *D. bryoniae* (strain DBJSJY2) was prepared as described above and was 10-fold serially diluted (from 10^5^ to 10^-2^ fg μL^-1^). The samples were then subjected to LAMP and PCR assays using the conditions described above. When the reactions were completed, the LAMP products were visualized as described above, while the PCR products were observed by 2.0% agarose gel electrophoresis. The LAMP and PCR assays were performed and assessed twice.

### Evaluation of the LAMP Assay Using Infected Plants

Muskmelon seedlings were inoculated with the two genotypes of *D. bryoniae* (strains DBJSJY2 and DBZJNB7; **Table 1**; **Supplementary Figure S1**). After 3 days, leaves showing suspected early symptoms were collected in greenhouse. The samples were prepared as described above. The LAMP assay was used to detect the presence or absence of *D. bryoniae* in muskmelon leaves using optimal assay temperatures and times. Healthy muskmelon plant leaves and ddH_2_O were used as negative controls, while purified DNA from *D. bryoniae* (strains DBJSJY2 and DBZJNB7) was used as a positive control. The reaction mixtures in the microcentrifuge tubes were visually inspected by the naked eye to determine the color change. The LAMP assay was performed and assessed twice.

## Results

### Optimization of the LAMP Assay

**FIGURE 2:**
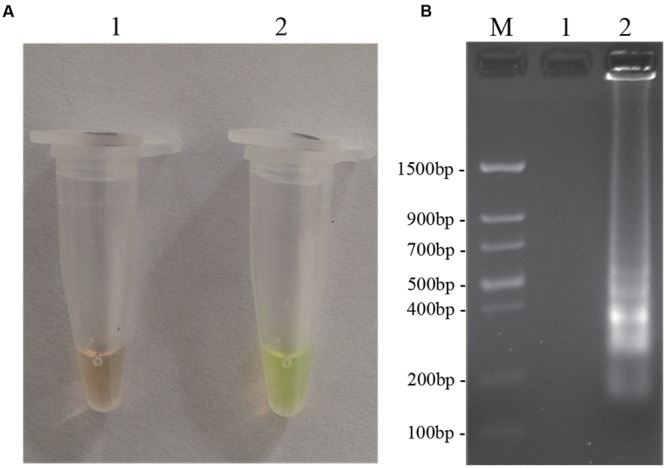
**LAMP detection of *D. bryoniae* (DBJSJY2).** Assessment is based on **(A)** LAMP for detection of *D. bryoniae* was using a fluorescence metal indicator (calcein) as a visual indicator. The positive reaction becomes yellowish-green, and the negative is still brown; **(B)** LAMP product was manifested as a ladder-like pattern on a 2.0% agarose gel. M: *Trans* DNA Marker II (Transgen Biotech, Beijing). In **(A,B)**, 1: Negative reaction (without target DNA), 2: Positive reaction (with target DNA). The same results were obtained in all three replicates.

**FIGURE 3:**
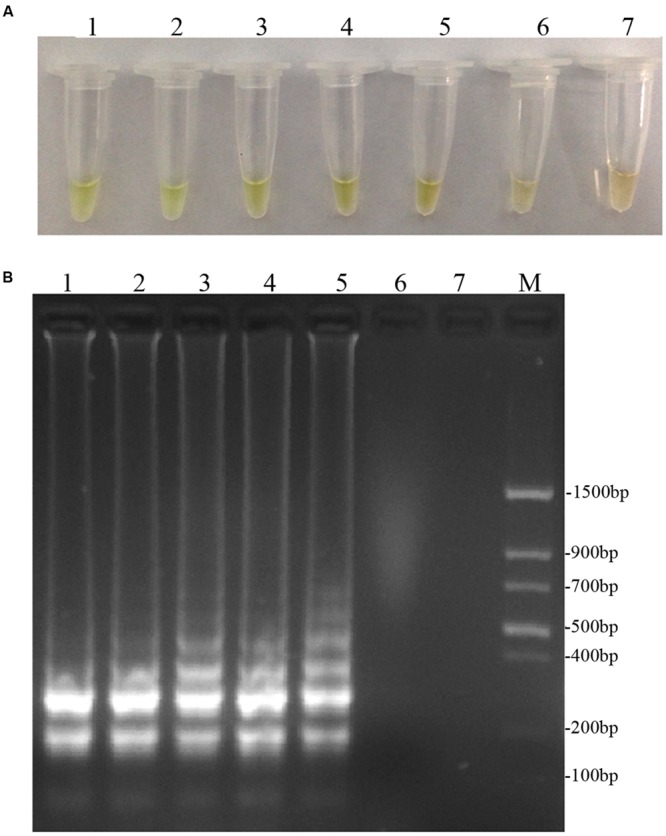
**Optimal reaction temperatures of LAMP. (A)** Detecting LAMP products by adding fluorescence metal indicator (calcein); the assessment was based on visualization of a color change from brown to yellowish-green. **(B)** Agarose gel electrophoresis analysis of the LAMP products. In **(A,B)**, lane 1: 61°C, lane 2: 62°C, lane 3: 63°C, lane 4: 64°C, lane 5: 65°C, lane 6: 66°C, lane 7: 68°C. M: *Trans* DNA Marker II (Transgen Biotech, Beijing). The same results were obtained in all three replicates.

**FIGURE 4:**
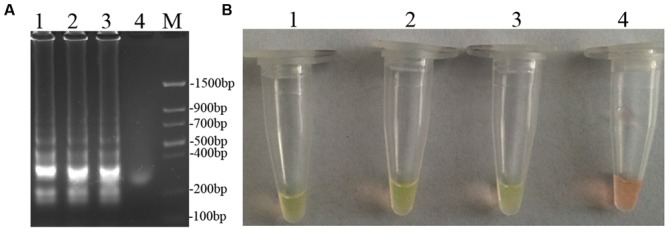
**Optimal reaction time of LAMP. (A)** Agarose gel electrophoresis analysis of the LAMP products. **(B)** Detecting LAMP products by adding a fluorescence metal indicators (calcein). In **(A,B)**, lane 1: 60 min, lane 2: 45 min, lane 3: 30 min, lane 4: 15 min, M: Trans DNA Marker II (Transgen Biotech, Beijing). The same results were obtained in two repeat assessments.

To optimize the efficiency of the LAMP reaction, the concentration of LAMP components was optimized using genomic DNA of *D. bryoniae* (strain DBJSJY2) as the template. The best results according to a fluorescence metal indicator (calcein; **Figure 2A**) and the typical ladder-like pattern on 2% agarose gel electrophoresis (**Figure 2B**) were obtained in a 25 μL volume containing 8 U *Bst* DNA polymerase, 2.5 μL 10x ThermoPol Buffer [New England Biolabs (Beijing) Ltd. Beijing, China], 8 mM MgSO_4_, 10 mM dNTPs, 4 μM each of DB17RG-FIP and DB17RG-BIP, 0.5 μM each of DB17RG-F3 and DB17RG-B3, 2 μM DB17RG-LB, 0.3 mM MnCl_2_, 8 μM calcein, and 1 μL target DNA. Based on the optimized reaction reagents, LAMP was performed using genomic DNA of *D. bryoniae* (strain DBJSJY2) as a template to determine the optimal temperature and reaction time. Positive results were obtained with temperatures of 61–65°C based on color change (**Figure 3A**) and the ladder-like pattern of the LAMP products (**Figure 3B**); however, the yellowish-green color intensity and ladder-like pattern of the LAMP products were strongest at 63°C. The positive reaction time in terms of color change (**Figure 4B**) and the ladder-like pattern of the LAMP products (**Figure 4A**) was as early as 30 min, but the yellowish-green color was more intense at 45 min than at 30 min. Thus, the optimal reaction conditions for the LAMP assay were 63°C for 45 min.

### Specificity of the LAMP Assay

**FIGURE 5:**
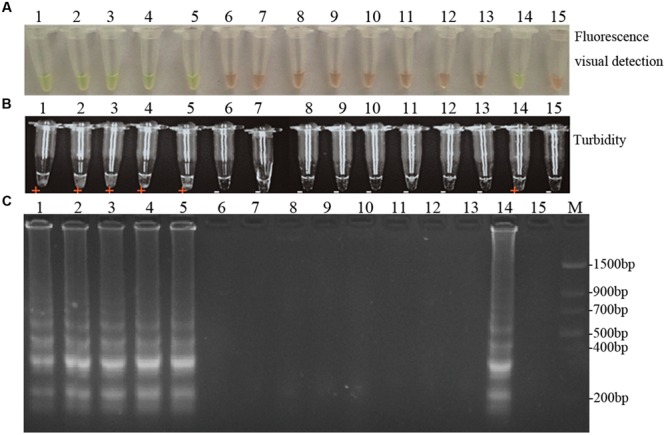
**Specificity of LAMP detection of *D. bryoniae*.** Assessment was based on **(A)** fluorescence metal indicator calcein visualization of color change, **(B)** the turbidity analysis of the LAMP products or **(C)** agarose gel electrophoresis analysis of the LAMP products. Lane 1, *Didymella bryoniae* (strain DBJSJY2) RGI; lane 2, *Didymella bryoniae* (strain DBAHHF2,) RGI; lane 3, *Didymella bryoniae* (strain DBZJNB5) RGI; lane 4, *Didymella bryoniae* (strain DBJSNJ60) RGI; lane 5, *Didymella bryoniae* (strain DBZJNB7) RGII; lane 6, *Ascochyta pinodes* ZJ-1; lane 7, *Colletotrichum orbiculare* NJ-1; lane 8, *Pythium paroecandrum Drechsler*; lane 9, *Alternaria alternata* LH1401; lane 10, *Fusarium verticillioide*; lane 11, *Fusarium oxysporum f.sp. niveum* Race 0; lane 12, *Fusarium oxysporum f.sp. niveum* Race 1; lane 13, *Fusarium oxysporum f.sp. niveum* Race 2; lane 14, positive control; lane 15, negative control. M, *Trans* DNA Marker II (Transgen Biotech, Beijing). The same results were obtained in two repeat assessments.

Using the optimal reaction conditions described above, and based on the presence of a PCR product of the expected size, the *D. bryoniae* strains used within this study were confirmed as RGI and RGII genotypes as shown in **Supplementary Figure S1** and **Table 1**. The LAMP assay was performed using DNA from fungal isolates (**Table 1**). As expected, the LAMP assay showed a yellowish-green color change and visible turbidity only for *D. bryoniae* strains, whether RGI or RGII genotype (**Figures 5A,B**). The assay showed high specificity in discriminating all *D. bryoniae* isolates from seven other fungal pathogens of Cucurbitaceae crops and *A. pinodes* (teleomorph: *Didymella pinodes*), a fungal pea pathogen, which is in the same genus as *D. bryoniae*. The typical ladder-like pattern of the LAMP products was obtained by agarose gel electrophoresis and confirmed the specificity of the LAMP assay (**Figure 5C**).

### Sensitivity of Detection among LAMP and Conventional PCR Assays

**FIGURE 6:**
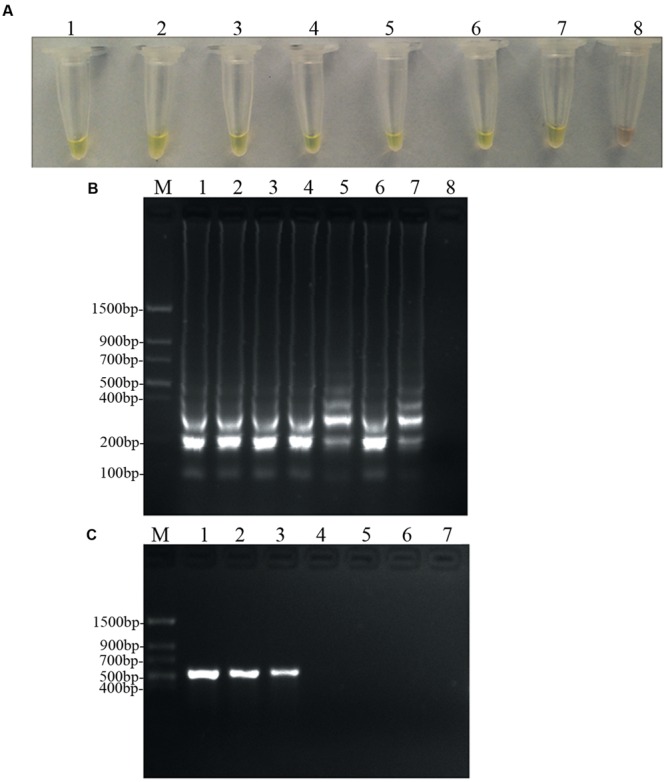
**Sensitivity of the LAMP and conventional PCR.** LAMP and conventional PCR assays using 10-fold serial dilutions of purified target DNA from *D. bryoniae* genomic DNA (strain DBJSJY2). **(A)** Detecting LAMP products by adding a fluorescence metal indicator (calcein). **(B)** Agarose gel electrophoresis analysis of the LAMP products. **(C)** Conventional PCR. Concentrations of template DNA (fg μL^-1^) per reaction in **(A,B)** were: lane 1 = 10^5^, lane 2 = 10^4^, lane 3 = 10^3^, lane 4 = 10^2^, lane 5 = 10, lane 6 = 1, lane 7 = 10^-1^ and lane 8 = 10^-2^. Concentrations of template DNA (fg μL^-1^) per reaction in **(C)** were: lane 1 = 10^5^, lane 2 = 10^4^, lane 3 = 10^3^, lane 4 = 10^2^, lane 5 = 10, lane 6 = 1 and lane 7 = 10^-1^. In **(B,C)**, M indicates a *Trans* DNA Marker II (Transgen Biotech, Beijing). The same results were obtained in two repeat assessments.

To determine and compare the detection limit, PCR and LAMP assays were performed using 10-fold serial dilutions of *D. bryoniae* genomic DNA. PCR products were detected by 2% agarose gel electrophoresis, and a 556 bp band specific for both genotypes of *D. bryoniae* could be seen (**Figure 6C**). As shown in **Figures 6A,B**, the limit of detection for the LAMP assay of genomic DNA of *D. bryoniae* was 0.1 fg μL^-1^ (**Figures 6A,B**), whereas the detection limit for conventional PCR was 100 fg μL^-1^ (**Figure 6C**). Thus, the LAMP assay was 1000-fold more sensitive than the conventional PCR. The same detection limits for the LAMP assay and for conventional PCR were obtained with four other *D. bryoniae* isolates (data not shown).

### LAMP Assay Using Infected Plants

**FIGURE 7:**
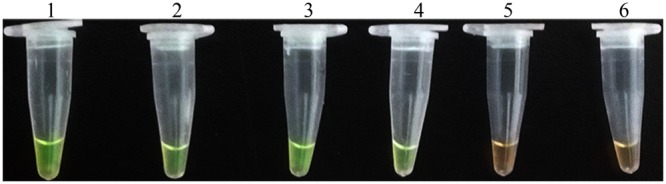
**LAMP detection of *D. bryoniae* from infected muskmelon leaves.** Lanes 1 and 2, purified DNA from *D. bryoniae* strains DBJSJY2 and DBZJNB7, respectively (positive controls); lanes 3 and 4, DNA from leaves infected with *D. bryoniae* strains DBJSJY2 and DBZJNB7, respectively; lane 5, control healthy muskmelon plant leaves; lane 6, ddH_2_O used as negative control. The same results were obtained in two repeat assessments.

Application of the LAMP assay for detection of *D. bryoniae* in infected muskmelon leaves was tested. Total DNA extracted from muskmelon samples was used as the template for the LAMP assay. All samples from seedling leaves with suspected primary symptoms reacted positively, but the samples from uninoculated leaves were negative (**Figure 7**). These results verified the potential field use of the LAMP assay.

## Discussion

Gummy stem blight caused by *D. bryoniae* is a highly prevalent disease and leads to significant losses in yield and quality on cucurbit crops worldwide (Keinath, 2011; Li et al., 2015). The occurrence of GSB disease in the greenhouse and in the field may arise from transplantation of latently infected source seedlings, or contamination by the pathogen may occur as a result of inappropriate pruning of unhealthy plants (Keinath, 1996, 2011; Ling et al., 2010). Thus, seedling health testing is a central issue for a large nursery. To control GSB of cucurbit crops, early crops with latent infection must be accurately detected and removed. Although very recently rapid detection of *D. bryoniae* by LAMP was reported (Tian et al., 2016), we found that the detection sensitivity and efficiency were lower in this method. In addition, research not has to investigate any infected samples under greenhouse or field conditions. In the current study, a credible, sensitive, and broad-spectrum diagnostic method for the detection of *D. bryoniae* isolates from pure cultures as well as from infected samples in the field was developed. The optimal reaction for detection of *D. bryoniae* could be carried out in less than 45 min and in a regular laboratory water bath that can provide isothermal conditions of 63°C. As LAMP assays have a high amplification efficiency, the LAMP method developed in this study showed a high sensitivity at 0.1 fg μL^-1^ of *D. bryoniae* genomic DNA, which was 1000-fold higher than that of a conventional PCR (**Figure 6**). The sensitivity was consistent with a previous report for LAMP methods used to detect *Sclerotinia sclerotiorum* (Duan et al., 2014a), and the sensitivity was greater than the recently reported LAMP method used to detect *D. bryoniae* (Tian et al., 2016). In addition, it has been reported that the LAMP reaction might be more efficient by using additional loop primers (Nagamine et al., 2002a; Parida et al., 2008). The optimal reaction time in our LAMP assay was shorter (i.e., less than 45 min) than that of Tian et al. (2016) for detection of *D. bryoniae*, who did not identify any suitable loop primers. One reason for this could be that in the present study we identified a suitable loop-backward primer and used the primer to accelerate the reaction. This improved the reaction time and efficiency. Studies have revealed that the time required for amplification with two loop primers is one-third to one-half of that without a loop primer, and amplification can be achieved within 30 min (Parida et al., 2008). However, due to the necessity of using a broad-spectrum diagnostic primer, we could not identify a suitable loop-forward primer, and the present LAMP assay only had one loop-backward primer. This appeared to result in the assay being slower than an assay that had both loop primers (Villari et al., 2013), and implies that we may be able to optimize the reaction time further.

To distinguish both genotypes of *D. bryoniae* from other fungal pathogens, a broad-spectrum specific LAMP primer set was designed based on a conserved sequence region (GenBank accession nos GQ872461 and GQ872462 (Ling et al., 2010);) common to both genotypes (RGI and RGII) of *D. bryoniae*. The specificity of the LAMP assay was confirmed using DNA of both genotypes of *D. bryoniae*, other fungal pathogens found in Cucurbitaceae crops and *A. pinodes* (teleomorph: *D. pinodes*), a fungal pea pathogen in the same genus as *D. bryoniae*. The result shown that the LAMP assay detected only the two genotypes of *D. bryoniae* and not for other fungal pathogens (**Figure 5**). Hence, this LAMP reaction using primer sets designed from the SCAR of genomic DNA for the two genotypes has high specificity and is broad-spectrum. Our result again supports the view that a LAMP assay can be widely used to diagnosis plant-pathogenic fungi (Denschlag et al., 2012; Niessen, 2015). Compared with the result of Ling et al. (2010), this improved broad-spectrum diagnostic method for *D. bryoniae* isolates was readily visible, easy to carry out and independent of PCR. Due to these advantages, the LAMP method is becoming more attractive to a wider range of users. Furthermore, to determine the utility of the LAMP assay, crude DNA isolated from infected muskmelon tissue samples was analyzed. All samples from muskmelon seedling leaves with suspected early symptoms reacted positively, but the samples from uninoculated leaves were negative (**Figure 7**). Similar results were found by Huang et al. (2011) and Shen et al. (2016) who used a LAMP assay to detect infected plant tissues. Compared with reported LAMP assays and conventional PCR, the LAMP assay reported here is more advantageous owing to its sensitivity and efficiency, and is robust enough to be used in latently infected crop testing applications in the field. These results indicate that the LAMP assay established in this study can be used for early detection of the disease as the detection limit is low, and the larger range for field use will significantly increase the efficiency of GSB diagnosis and management. Consequently, it is worth emphasizing that the advantages offered by this LAMP assay provide a robust, visual, and easy-to-perform approach for detecting *D. bryoniae* in early crops. Therefore, this assay could be useful even for amateur farmers without the need for elaborate laboratory equipment. The potential application of this diagnostic tool will enable early prediction of disease, reducing the risks of epidemics.

LAMP-based assays are growing in popularity, and have been applied to the detection of many plant-pathogenic fungi (Niessen, 2015). However, the use of a real-time turbidimeter is not applicable in rural areas of developing countries. Thus, for the diagnosis method to be used at the agricultural site, a simple, reliable, and unambiguous visual inspection method is required. As the LAMP reaction progresses, pyrophosphate ions are produced as the byproduct of the reaction and bind to divalent cations (Mn^2+^ or Mg^2+^). Monitoring of the products is easy by the naked eye using DNA-binding dyes such as SYBR Green after the reaction endpoint (Huang et al., 2011; Chandra et al., 2016). However, use of DNA-binding dyes increases the rates of cross-contamination because of the open cover operation (Notomi et al., 2000; Zhang et al., 2014). To avoid this, visualization of indirect colorimetric indicators such as hydroxynaphthol blue or calcein have been used as an improvement (Niessen and Vogel, 2010; Duan et al., 2014b). Here, we added calcein as an indirect indicator before the reaction; calcein molecules combine with Mn^2+^ and quench calcein fluorescence. If the LAMP reaction is positive, the calcein molecules release Mn^2+^ and combine with residual Mg^2+^ to generate pyrophosphate ions, thereby recovering their green fluorescence signal (Zhang et al., 2014). Hence, compared with the two-step DNA-binding dye approach, the risk of cross-contamination is much lower (Parida et al., 2008; Duan et al., 2014b; Zhang et al., 2014). A positive reaction is indicated by a color change from brown to a yellowish-green color. In this study, the positive and negative reactions could be successfully distinguished with the naked eye by adding calcein. The fluorescent signals clearly correlated with the results of analysis by gel electrophoresis (**Figure 2**).

Although the LAMP assays in this study showed high specificity and sensitivity, and showed the highest detection limit so far in the subfemtogram range (Niessen, 2015), we still need to be cautious because the presence of calcein may inhibit the LAMP reaction and reduce the sensitivity (Wastling et al., 2010; Zhang et al., 2012). In addition, although the developed LAMP assay showed high sensitivity and broad-spectrum detection of *D. bryoniae* isolates, we only tested samples by artificial inoculation of muskmelon in the greenhouse. Thus, further studies need to be carried out on a larger scale, and more field samples from other Cucurbitaceae crops in addition to muskmelon should be used to confirm the specificity of the assay.

## Conclusion

A visual LAMP method has been developed for rapid and broad-spectrum detection of *D. bryoniae* with the advantages of simplicity, sensitivity, and specificity. The LAMP assay established in this study can be used for numerous applications, such as the potential field use for efficient GSB diagnosis and management, seed quarantine, and evaluation of GSB resistance in breeding procedures. The prospective application of this diagnostic tool for early and accurate detection of the causal agent of GSB in cucurbit crops could also lead to reduced fungicide use, thus benefiting the environment. Hence, this study represents a successful attempt to develop a LAMP-based detection method of infection in early cucurbit crops.

## Author Contributions

Conceived and designed the experiments: XfY and XpY. Performed the experiments: XfY, PL, and MZ. Analyzed the data: XpY, RR, GL, and JX. Wrote the paper: XfY and XpY.

## Conflict of Interest Statement

The authors declare that the research was conducted in the absence of any commercial or financial relationships that could be construed as a potential conflict of interest.

## Footnotes

1 http://primerexplorer.jp/elamp4.0.0/index.html

## Supplementary Material

FIGURE S1 **Assignment to RGI and RGII genotypes of *D. bryoniae* strains by RG-specific PCR.** DNA of *D. bryoniae* isolates amplified with the *D. bryoniae* RGI-specific primers **(A)** and RGII-specific primers **(B)**. Lane 1, *Didymella bryoniae* (DBJSJY2) RG I; lane 2, *Didymella bryoniae* (DBAHHF2) RG I; lane 3, *Didymella bryoniae* (DBZJNB5) RG I; lane 4, *Didymella bryoniae* (DBJSNJ60) RG I; lane 5, *Didymella bryoniae* (DBZJNB7) RG II; M indicates a DS200 marker and marker 1 (Dongsheng biotech, Guangzhou).
