# Supplementary figures and images for: Rapid and Sensitive Detection of Didymella bryoniae by Visual Loop-Mediated Isothermal Amplification Assay
Source: Front Microbiol. 2016 Aug 30;7:1372. doi: 10.3389/fmicb.2016.01372 (PMC5003822; doi:10.3389/fmicb.2016.01372)

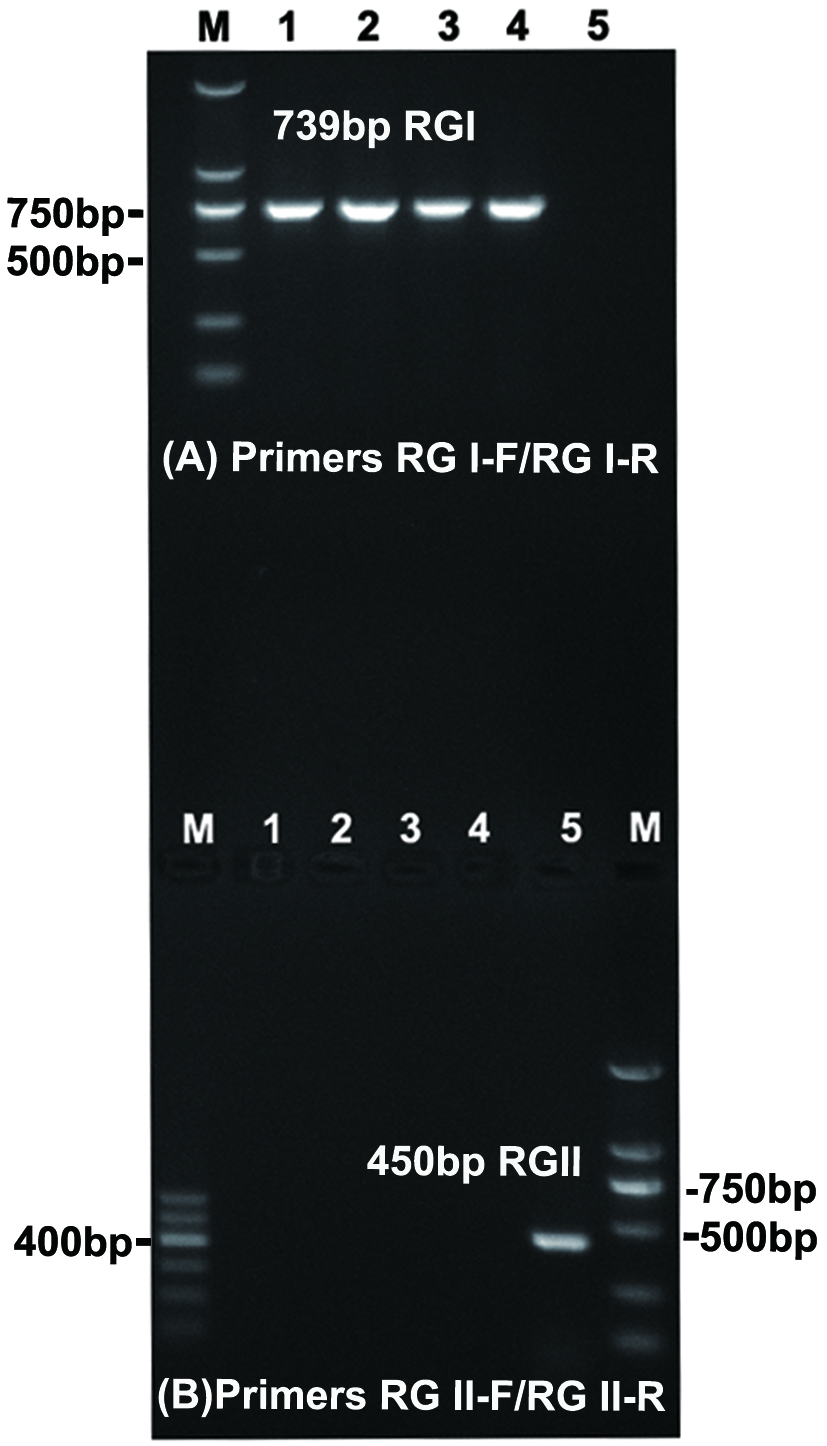

Supplement: FIGURE S1 — Assignment to RGI and RGII genotypes of D. bryoniae strains by RG-specific PCR. DNA of D. bryoniae isolates amplified with the D. bryoniae RGI-specific primers (A) and RGII-specific primers (B). Lane 1, Didymella bryoniae (DBJSJY2) RG I; lane 2, Didymella bryoniae (DBAHHF2) RG I; lane 3, Didymella bryoniae (DBZJNB5) RG I; lane 4, Didymella bryoniae (DBJSNJ60) RG I; lane 5, Didymella bryoniae (DBZJNB7) RG II; M indicates a DS200 marker and marker 1 (Dongsheng biotech, Guangzhou). [file Image_1.TIF]
